# Protein-based micro- and nano-transporters for gut microbiota modulators: advances in delivery strategies and gut health benefits

**DOI:** 10.3389/fbioe.2025.1706245

**Published:** 2025-10-13

**Authors:** Mohammad Nasiruddin Rana, Md. Saifullah, Abu Saeid, Md. Murtuza Kamal, Xiangjing Gao, Xiangwei Gao

**Affiliations:** ^1^ Department of Clinical Laboratory of Sir Run Run Shaw Hospital, School of Public Health, Zhejiang University School of Medicine, Hangzhou, China; ^2^ Centre for Food Innovation, Tasmanian Institute of Agriculture, University of Tasmania, Newnham, TAS, Australia; ^3^ Department of Biochemistry, Memorial University of Newfoundland, St. John’s, NL, Canada; ^4^ Department of Food Processing and Preservation, Hajee Mohammad Danesh Science and Technology University, Dinajpur, Bangladesh; ^5^ Department of Occupational Health and Radiation Protection, Zhejiang Provincial Center for Disease Control and Prevention, Hangzhou, China

**Keywords:** gut microbiota, gut disorders, probiotics, bioactive phytochemicals, protein-based transporters

## Abstract

Human gut microbiota (GM) is essential for gut health, and its dysbiosis is associated with diseases such as inflammatory bowel disease, obesity, and cancers. Natural GM modulators, including probiotics, bioactive phytochemicals, and peptides, have shown great potential in restoring microbial balance. However, their effectiveness is limited by stability, bioavailability, and targeted delivery to the gut. Protein-based micro- and nano-transporters have recently emerged as innovative platforms capable of overcoming these delivery barriers and improving the therapeutic performance of natural GM modulators. Here, we critically review the design strategies, functional performance, and therapeutic outcomes of various protein-based carriers, including composite hydrogels, stimuli-responsive microgels, targeted nanocomplexes, mucoadhesive microcapsules, and electrospun nanofibers. Furthermore, we discuss how these advanced systems ameliorate gut dysbiosis, modulate immune responses, enhance intestinal barrier integrity, and promote beneficial microbial growth. Finally, current challenges and future perspectives for clinical translation are outlined. Overall, this review aims to provide a mechanistic and practical foundation for the development of next-generation protein-based transporters for microbiota-targeted therapies.

## 1 Introduction

The human gut microbiota (GM), composed of bacteria, archaea, bacteriophages, viruses, and fungi, plays essential roles in digestion, nutrient absorption, metabolism, immune regulation, and neural signaling (Nicolas and Ma, 2025). *Firmicutes, Bacteroidetes, Actinobacteria,* and *Proteobacteria* are the four phyla that represent >90% of the total GM, with *Firmicutes* accounting for the majority (50%–75%), followed by *Bacteroidetes* (10%–50%), *Actinobacteria* (1%–10%), and *Proteobacteria* (<1%) (Guan et al., 2025). By interacting with immune cells and producing metabolites such as short-chain fatty acids (SCFAs), the GM supports energy balance, maintains intestinal barrier function, and exerts anti-inflammatory and anti-cancer effects (Li Y. et al., 2024). Disruption of this ecosystem, termed gut dysbiosis, is characterized by loss of beneficial microbes, overgrowth of pathogens, and reduced diversity. Dysbiosis contributes to diseases including diarrhea, inflammatory bowel disease (IBD), obesity, type 2 diabetes, and colorectal cancer (Nicolas and Ma, 2025). A recent case study on the worldwide burden of digestive diseases in 204 countries from 1990 to 2019 demonstrated that digestive diseases are a major contributor to the global healthcare burden, with age-standardized incidence cases of 7.3 billion and prevalent cases of 2.9 billion, accounting for more than one-third of prevalent disease cases and approximately one-fifth of incident disease cases, highlighting the urgent need for effective interventions (Wang et al., 2023). These findings have sparked interest in altering GM as a possible strategy for treating gut disorders and associated diseases.

**FIGURE 1:**
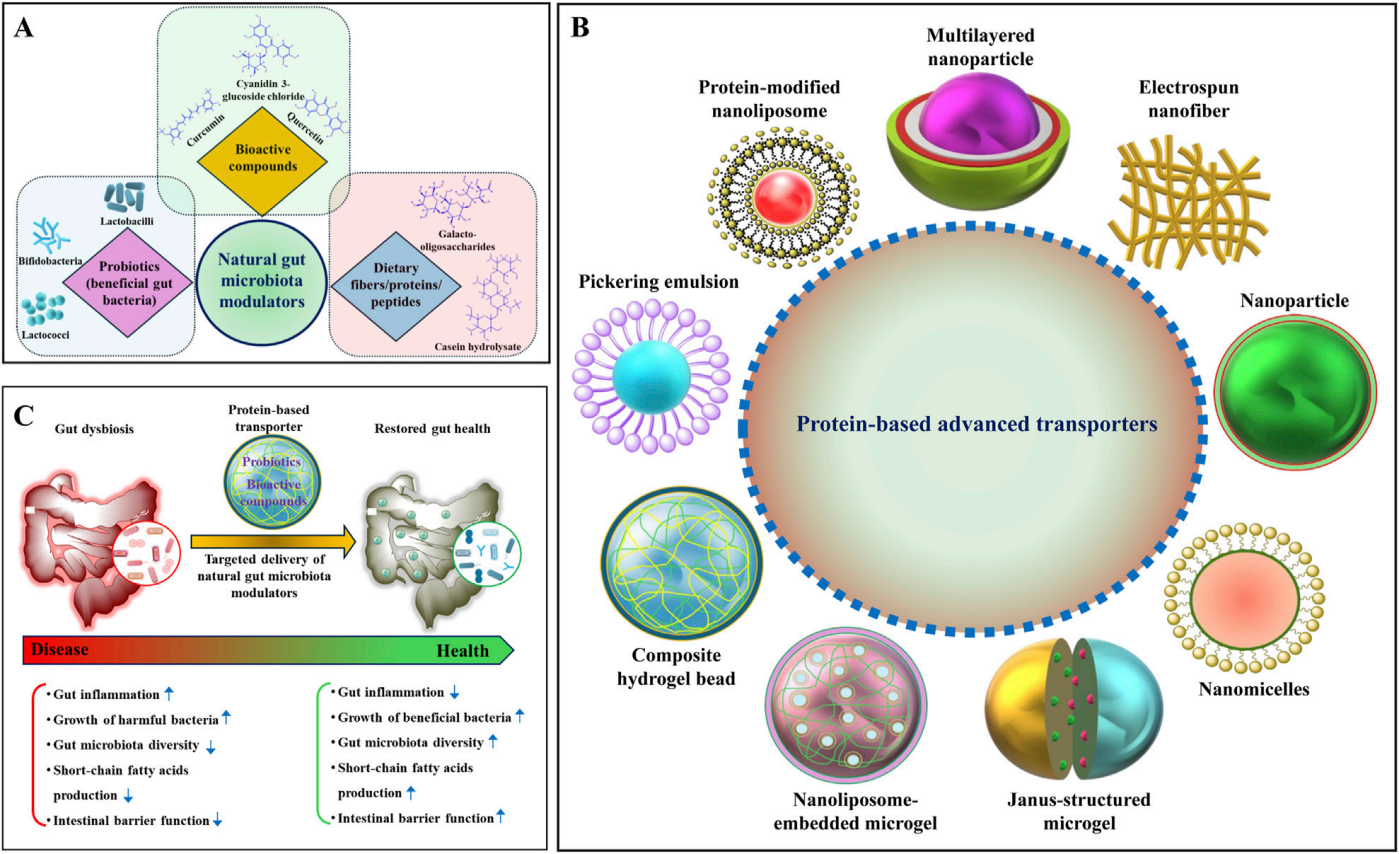
Schematic overview of natural gut microbiota (GM) modulators, protein-based transporters, and their role in alleviating dysbiosis. **(A)** A schematic illustration of various natural GM modulators, including bioactive compounds, probiotics, dietary fibers, and proteins/peptides. **(B)** Various protein-based advanced transporters have been developed for the delivery of natural GM modulators. **(C)** A hypothetical illustration of reducing gut dysbiosis by the supplementation of natural GM modulators using a protein-based transporter.

Natural GM modulators—including probiotics, bioactive phytochemicals, dietary fibers, and peptides—can restore microbial balance via antioxidant and anti-inflammatory activity, suppression of pathogenic bacteria, and regulation of immune homeostasis (Figure 1) (Cheng et al., 2024; Mao et al., 2024; Guan et al., 2025). However, some of these natural modulators exhibit low bioavailability due to degradation in the harsh gastrointestinal environment, poor solubility, or insufficient colon-targeted release. Probiotics are living bacteria (e.g., *Lactobacillus rhamnosus*) that play an important role in modifying GM, but they cannot survive in high-acid gastric conditions (Han et al., 2025). Similarly, bioactive compounds like quercetin and curcumin can be readily destroyed in the gastrointestinal (GI) tract before reaching the target location or physiological absorption for metabolic activity (Bai et al., 2025; Liu et al., 2025a). On the contrary, protein-based transporters offer an attractive solution owing to their biocompatibility, biodegradability, mucoadhesiveness, and stimuli-responsiveness. They provide a promising strategy for protecting active components, targeting inflamed tissues, and offering controlled-release, making them superior to traditional systems and paving the way for next-generation gut therapeutics (Jiang et al., 2024; Murphy et al., 2025). To maximize the potential of these advanced transporters, it is crucial to comprehend their mechanism and therapeutic efficacy in GI disorders.

To date, several reviews have addressed drug delivery systems, such as protein/peptide-based systems for renal targeting, protein/polysaccharide-based systems for targeted delivery of polyphenols and carotenoids, and albumin-based delivery systems (Jiang et al., 2024; Lu et al., 2024; Murphy et al., 2025). However, existing reviews offer limited coverage of recent advances in protein-based transporters, particularly with respect to their biological performance and therapeutic efficacy in alleviating gut-related disorders. Therefore, this review summarizes state-of-the-art protein-based micro- and nano-transporters for the intestine-specific delivery of natural GM modulators. We focus on their design, delivery efficiency, and therapeutic performance based on *in vitro*, cellular, and preclinical studies, and further discuss current challenges and potential solutions.

## 2 Mechanism of natural GM modulators in promoting gut health

### 2.1 Role of bioactive compounds

**FIGURE 2:**
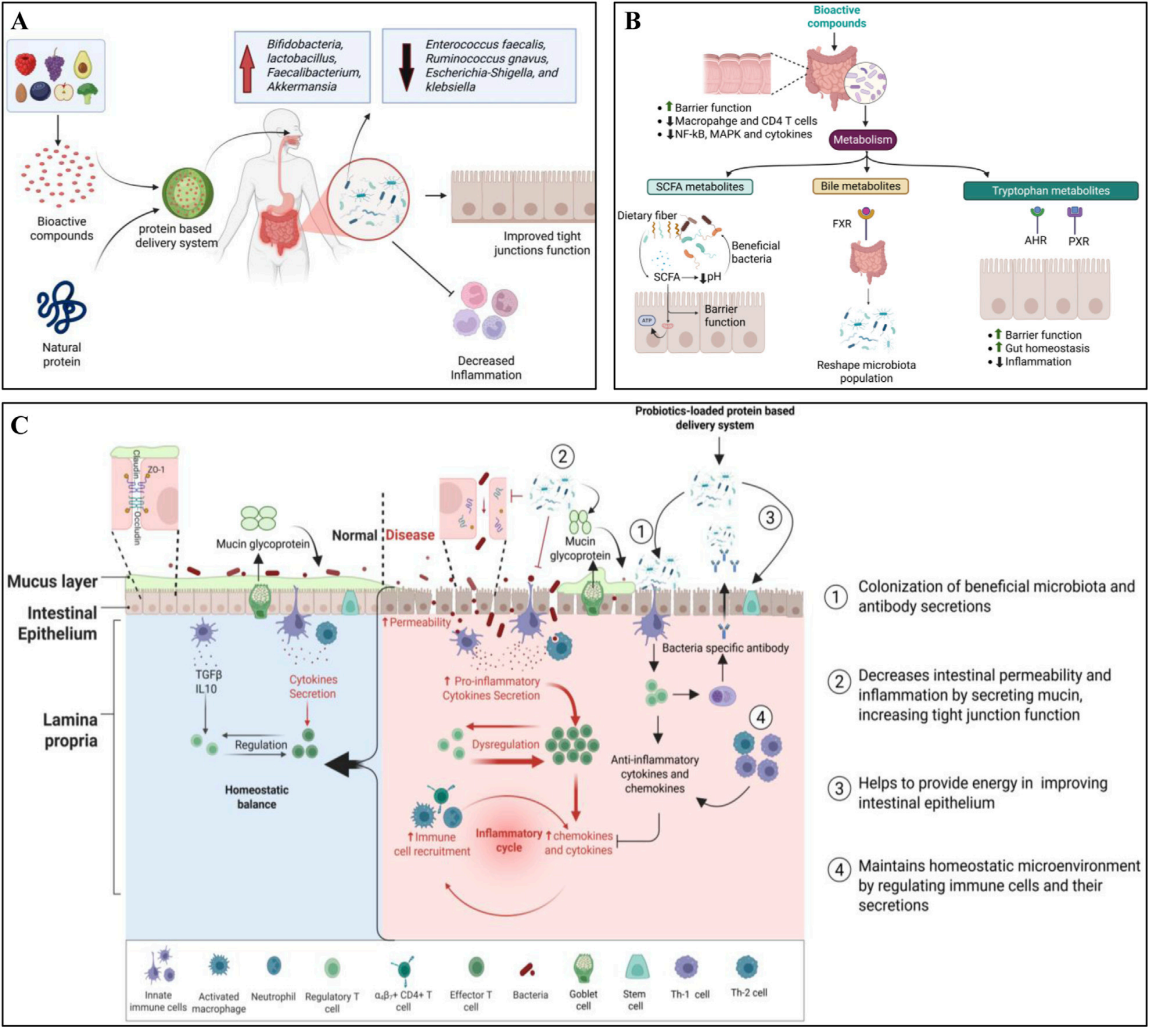
Mechanisms of natural GM modulators in promoting gut health. **(A)** Natural GM modulators-loaded protein-based transporters for improving gut health. **(B)** Role of bioactive molecules in regulating gut barrier function, gut homeostasis, and inflammation. **(C)** Role of probiotics-loaded protein-based transporters in improving immune function, gut barrier function, and gut homeostasis.

Bioactive substances can play a crucial role in promoting gut health by altering GM through several mechanisms: altering microbial composition, regulating metabolite production, and modulating the immune system (Gowd et al., 2019). After protective and controlled delivery using protein-based nano-transporters, bioactive molecules, e.g., quercetin, curcumin, resveratrol, and protein peptides, can promote the growth of beneficial bacteria (*Bifidobacteria, Lactobacilli,* and *Faecalibacterium, Akkermansia* species, etc.), and suppress the growth of harmful bacteria (*Enterococcus faecalis, Ruminococcus gnavus, Escherichia-Shigella,* and *Klebsiella*, etc.) to maintain the gut microbial homeostasis, which in turn reduces the intestinal inflammation and strengthens the intestinal barrier (Figure 2A) (Yang J. et al., 2024; Liu et al., 2025a; Ma S. et al., 2025). They can also inhibit harmful bacteria by affecting bacterial enzymes, disrupting cell walls and membranes, and altering bacterial communication. For instance, catechins can inhibit bacterial DNA gyrase and dihydrofolate reductase, which are responsible enzymes for bacterial DNA replication and folate synthesis, respectively (Reygaert, 2018). In addition, bioactive compounds, proteins, and dietary fibers can serve as a prebiotic substrate for microbial metabolism to produce useful metabolites like SCFAs, which lower the colonic pH, assist the growth of beneficial bacteria, and inhibit the growth of pathogenic bacteria (Yuan et al., 2022; Guo et al., 2023). They can modulate host immune responses by influencing immune cell functions and cytokine production. For instance, curcumin and quercetin downregulate pro-inflammatory cytokine production by modulating NF-κB and MAPK signaling pathways in the intestine, reduce gut inflammation, and protect the gut barrier by maintaining the integrity of tight junctions (Bai et al., 2025; Sun et al., 2025). Several bacterial metabolites, including SCFAs, bile acid metabolites, tryptophan metabolites, phenolic metabolites, and polyamines, interact with host receptors (e.g., GPR41, GPR43, TGR5, FXR, etc.) to regulate immune responses, suppress inflammation, improve intestinal barrier function, and maintain gut homeostasis (Figure 2B) (Liu et al., 2022). Therefore, dietary intervention of bioactive compounds loaded into advanced transporters represents a promising strategy for maintaining gut health and managing and/or preventing a wide range of diseases, including IBD, colorectal cancer, cardiovascular diseases, and metabolic syndromes via restoring GM balance.

### 2.2 Role of probiotics

Probiotics can play a crucial role in modulating GM by enhancing microbial diversity, promoting beneficial bacteria, and suppressing pathogenic species. Probiotics-mediated GM modulation helps to improve metabolic health, immune function, and disease prevention following multiple mechanisms (Chandrasekaran et al., 2024). After protective intestinal delivery, probiotics increase the abundance of beneficial bacteria, including *Bifidobacterium, Lactobacillus, Bacillus,* and *Eubacterium*, while they reduce the harmful genera, e.g., *Enterococcus, Fusobacterium,* and *Pseudomonas*. This modulation enhances the fermentation process of prebiotics to increase the production of SCFAs, which in turn improve intestinal homeostasis and integrity of the gut barrier, and reduce inflammation and gut dysbiosis-associated complexities (Chandrasekaran et al., 2024). Probiotic treatment counteracts pathogenic microorganisms by producing antimicrobial substances, such as bacteriocins, organic acids, etc. For example, *Bacillus* species produce fengycins (an antifungal lipopeptide) to inhibit *Staphylococcus aureus* via inhibiting quorum sensing (Kamada et al., 2012). On the barrier and immune fronts, probiotics stimulate mucin secretion, upregulate tight junction proteins, and strengthen epithelial cohesion, thereby reducing translocation and permeability (Ma et al., 2023). Immunomodulatory effects include activation of antigen-presenting cells, expansion of regulatory T cells, balancing of Th1/Th2 responses, and increased production of anti-inflammatory mediators such as IL-10 and TGF-β, culminating in attenuated mucosal inflammation and improved host–microbiome homeostasis (Figure 2C) (Liu et al., 2021; Chandrasekaran et al., 2024).

In conclusion, both bioactive compounds and probiotics operate through complementary, microbiota-dependent and -independent pathways to restore gut function. Crucially, the efficacy of these modulators is contingent on delivery performance; protein-based transporters that preserve cargo integrity, control release kinetics, and promote site-specific action are therefore central to translating microbiome-targeted interventions into reliable therapeutic and preventive strategies. The following sections critically evaluate the delivery performance and gut-health outcomes associated with different protein-based transporter platforms.

## 3 Gelatin-based transporters

### 3.1 Chemical and functional properties of gelatin

Gelatin is a natural polypeptide polymer derived from the partial hydrolysis of collagen using acid, base, or enzymatic treatments found in animal connective tissues, bones, and skins (Mohanto et al., 2023). It is a colorless, odorless, high-protein, low-energy substance consisting of 18 amino acids, including glycine, proline, and hydroxyproline. Due to the coexistence of both positive and negative charges as well as hydrophilic and hydrophobic groups, gelatin behaves as a polyampholyte and amphiphilic macromolecule. These properties enable it to interact with oppositely charged biopolymers and to adsorb at lipid–aqueous interfaces, making it highly versatile in biopolymer complexation (Tan et al., 2023).

The physicochemical properties of gelatin vary with the type of treatment applied to collagen. For example, gelatin derived through acid hydrolysis exhibits higher gel strength with an isoelectric point (PI) at a pH of 7.0–9.5, known as type-A gelatin, while the basic hydrolyzed gelatin known for its weak gel strength with a PI at a pH of 4.7–5.2, known as type-B gelatin (Tan et al., 2023). Additionally, fish-derived gelatin typically exhibits weaker gel strength than mammalian sources (Kaynarca et al., 2025). Varying PI and gelling strength influence its intended use. Owing to its biocompatibility, biodegradability, film- and gel-forming ability, and cost-effectiveness, gelatin has been widely applied in bioactive compound encapsulation and transporter development (Mohanto et al., 2023). However, electrolyte status, high hydrophobicity, pH variation, and thermal treatment can influence its viscosity and gelling capacity. Nevertheless, enzymatic and chemical crosslinking with other biopolymers can improve its physicochemical stability and exert great potential in delivering bioactive compounds (Mohanto et al., 2023; Tan et al., 2023).

### 3.2 Delivery performance and gut health improvements of gelatin-based transporters

Gelatin has been extensively explored as a carrier for the controlled delivery of bioactive compounds and probiotics to the gastrointestinal tract (Table 1). For example, Wu et al. used four selective wall materials, namely, gelatin, soy protein isolate, maltodextrin, and gum Arabic (GA), for the delivery of blueberry anthocyanins and modulating microbial flora composition (Wu et al., 2020). As a single polymer, both proteins were efficient in improving colonic accessibility, delaying anthocyanin release, and promoting intestinal health compared to other carrier materials. The microcapsule formulations enhanced the quantity and composition of *Bacteroidetes* while decreasing that of *Firmicutes*. In particular, the protein-based anthocyanin formulation promoted the production of more phenolic compounds and SCFAs, resulting in enhanced antioxidant activity during *in vitro* colonic fermentation (Wu et al., 2020). Nevertheless, gelatin alone is highly susceptible to proteolytic enzymes (e.g., pepsin, trypsin), which can cause premature release in the upper GI tract. To improve stability, gelatin is often combined with polysaccharides. For instance, gelatin–carboxymethyl cellulose complexes enhanced the thermal stability of zeaxanthin, enabling sustained gastric release and maximal intestinal delivery (Zhang J. et al., 2021).

**TABLE 1:** Protein-based transporters and their application for the delivery and modulation of gut health *In vitro* and *in vivo* studies.

Type of protein	Transporter system	Formation mechanism and active compounds	Key features of transporter *In vitro* studies	Effects on gut health *In vivo* studies	References
Gelatin	ROS-responsive dopamine-coated gelatin nanoparticle (Gel/DA-NPs)	• Self-assembly and polymerization Quercetin (Q)	• Q/Gel/DA-NPs had uniform size distribution, good physical stability, antibacterial effects, and antioxidant properties • They extended resident time in the gastrointestinal (GI) tract and enhanced bioavailability of Q The dopamine shell showed ROS-responsive degradation upon reaching the colon lesion	• Q/Gel/DA-NPs significantly alleviated the ulcerative colitis (UC) symptoms in a dextran sodium sulfate (DSS)-induced mouse model • It enhanced body weight, reduced fecal occult blood, and restored compromised crypt structures It reduced inflammation by promoting the polarization of M2 macrophages to reduce apoptosis and modulate immunity	Bai et al. (2025)
Gelatin	“Janus” structured nanoclay microgel	• Ionic gelation *Pediococcus pentosaceus* Li05	• The resultant nanoclay microgel was stable in gastric fluid and releases probiotics in intestinal fluid • It significantly enhanced the oral bioavailability of probiotics and exhibited targeted delivery of active probiotics to the intestines	• The nanoclay microgel achieved significant therapeutic effects in a rat model of IBS-D • It improved weight loss, diarrhea, and visceral hypersensitivity • It inhibited the secretion of inflammatory factors by suppressing the NLRP3 inflammasome pathway It repaired intestinal mucosal damage and modulated the dysregulated intestinal microbiota	Wu et al. (2025)
Soy protein isolate (SPI)	Highly adhesive bilayer microcapsule (LP@TAFe/SX)	• Ligand bonding, electrostatic interaction, and hydrogen bonding *Lactiplantibacillus plantarum* KLDS1.0386 (LP)	• The adhesion rate of LP was enhanced up to 16.7-fold with increasing tannic acid (TA) levels • The addition of SPI-xanthan gum complex (SX) to the microcapsule resulted in a rougher surface, a greater negative surface charge, and larger particles, indicating the existence of an SX layer and LP encapsulation The SX layer improved GI tolerance and increased the survival rate of LP by up to 8.5 times compared to free LP.	• The LP@TA-Fe/SX therapy reduced colonic tissue damage and epithelial inflammation and repaired the compromised intestinal barrier It restored the imbalanced GM by lowering pathogens and boosting probiotic bacteria (*Lachnospiraceae*_NK4A136_group, *Muribaculaceae*)	Zhao et al. (2025)
Soybean 7S protein	Soybean 7S/calcium phosphate complexes (7S/CaP)-based tablet	• Ionic crosslinking and tablet press *Lactobacillus rhamnosus*	• The 7S-0.8%/CaP combination was the most effective formulation for protecting lactic acid bacteria in tablets The 7S/CaP complex tablets protected *Lactobacillus rhamnosus* throughout gastric digestion while also allowing for the regulated release of probiotics during intestinal digestion	• The probiotic tablet treatment protected the colonic epithelium from pathological damage, restored the intestinal epithelial barrier, and increased the GM diversity in DSS-induced mice • It presents a novel strategy for developing a synbiotic-based therapeutic formulation to treat gastrointestinal issues	Han et al. (2025)
Whey protein isolate (WPI)	WPI/galactooligosaccharides (GOS)/fructooligosaccharides (FOS) conjugates	Glycosylation	• WPI/GOS conjugates exhibited a deeper glycation extent and more pronounced structural unfolding and helix-destabilizing compared to WPI/FOS conjugates WPI/GOS conjugates improved functional indicators of RAW264.7 macrophages	• In immunosuppressed mice, WPI/GOS conjugates repaired the immune organ and intestinal barrier as well as increased IL-1β and IFN-γ levels • WPI/GOS conjugates modulated GM by upregulating *Lactobacillus* and changed the serum metabolites, which activated the NFκB pathway and strengthened the immune system WPI samples reduced the crypt depth, elevated the villus length/crypt depth ratio, and restored the serous layer	Mao et al. (2024)
Whey protein isolate (WPI)	WPI/*Tremella fuciformis* polysaccharides (WPI/TFPS)-stabilized Pickering emulsion	• Electrostatic complexation and emulsification Magnolol (Mag)	• Mag/WPI/TFPS formed stable microparticles with excellent encapsulation efficiency (93.63%) It exhibited sustained-release properties in simulated colon fluid with a cumulative release rate of 60.78% at 48 h	• Mag/WPI/TFPS accumulated in the colon tissue for 24 h with stronger fluorescence intensity • It alleviated the symptoms of DSS-induced UC mice, e.g., increased the colon length and decreased the DAI score • It protected the colon tissue by improving the crypt structure, enhancing the tight junction proteins (ZO-1 and occludin), and lessening the inflammatory factors (TNF-α and IL-6) WPI/TFPS treatment enhanced the GM balance by increasing the diversity and relative abundances of *Lactobacillaceae* and *Firmicutes*	Zhang et al. (2024)
Casein	Curcumin/egg white peptide aggregates (Cur/EWP) and curcumin/egg white peptide/casein/quaternary chitosan complexes (Cur/EWP/CA/QC)	• pH-driven self-assembly and electrostatic complexation EWP and curcumin	• Both Cur/EWP and Cur/EWP/CA/QC complexes demonstrated excellent colloidal stability and biocompatibility • The nano-complexes were uniform, regular, spherical, and around 156.0 nm and 300.0 nm, respectively The curcumin content was ∼66.4% in EWP and ∼86.4% in EWP/CA/QC after simulated intestinal digestion, indicating excellent curcumin protection against adverse GI conditions	• In DSS-induced UC mice, Cur/EWP/CA/QC showed synergistic efficacy by reducing DAI and colonic damage • It suppressed the levels of colonic inflammatory cytokines (TNF-α, IL-1β, and IL-6) and increased the level of anti-inflammatory cytokine (IL-10) • It promoted the growth of beneficial bacteria (*Muribaculaceae* and *Bacteroidaceae*) and enhanced the production of SCFAs It enhanced the permeability of Cur and EWP in the small intestine, which activated immunological response and enhanced the tight junction proteins (ZO-1 and MUC-2)	Li et al. (2024d)
Casein phosphopeptide (CPP)	Tannic acid/Mg^2+^ and CPP complexes (TA/Mg^2+^@CPP)	• Coordination bonding and Layer-by-layer coating *Saccharomyces boulardii* (SB)	• SB@TA/Mg^2+^@CPP improved the GI stability It demonstrated strong cell viability and antioxidant activity	• SB@TA/Mg^2+^@CPP decreased the phenotypic alterations of colons and improved the abundance and diversity of GM (increased beneficial bacteria, e.g., *Muribaculaceae*, and decreased the pathogenic bacteria, e.g., *Escherichia-Shigella*) It increased tight junction proteins (ZO-1 and occludin), reduced inflammatory parameters, and promoted magnesium adsorption in DSS-induced colitis mice	Qian et al. (2025)
Zein	Egg white-derived peptide (EWDP) -co-assembled zein/chondroitin sulfate nanoparticle	• Layer-by-layer co-assembly Quercetin	• The NPs demonstrated exceptional pH-sensitive size tunability (488.0–128.6 nm), quercetin EE (99.3%), and GI stability EWDP may operate as prospective co-assembly enhancers and excipient analogs to produce superior oral synergism (e.g., antioxidant and anti-inflammatory)	• NPs efficiently repaired intestinal tight junctions and mucus layers and restored GM composition by encouraging beneficial bacteria and reducing pathogens in the gut This work offers a simple and effective oral colon-targeted nano-strategy for various nutraceuticals in targeted UC alleviation	Ma et al. (2025b)
Zein	Zein/soluble soybean polysaccharide nanoparticles (ZPS)	• Antisolvent precipitation *Bacillus subtilis*	• ZPS nanoparticles synthesized at pH 6.0 demonstrated higher encapsulation efficiency (EE) for *B. subtilis* Encapsulated probiotics had a significantly higher survivability of 3.13, 3.20, and 1.50 times that of free probiotics after GI digestion, pasteurization, and storage	• Probiotics encapsulated in ZPS suppressed the abundance of potentially pathogenic bacteria (*Escherichia-Shigella*) It enhanced the makeup of the GM, microbial diversity, and the abundance of good bacteria (*Muribaculaceae*, *Romboutsia, Bifidobacterium*, and *Lactobacillus*)	Cheng et al. (2024)
Chickpea protein	Chickpea protein isolate/citrus pectin conjugate-stabilized O/W emulsion (CPI/CP emulsion)	• Glycosylation and emulsification Curcumin	• The CPI/CP emulsion exhibited improved curcumin stability in the presence of heat, UV radiation, and oxidation, with a high curcumin EE of 86.15% It improved intestine-targeted delivery and increased curcumin bioavailability by up to 85.60%	• The CPI/CP emulsion was biologically safe and effective in reducing liver oxidative stress and improving gut health Emulsion treatment enhanced SCFAs production, improved good bacteria like *Firmicutes*, and lowered detrimental microbes, e.g., *Helicobacteraceae*, indicating its ability to alter GM composition	Liu et al. (2025a)
Lactoferrin	Melatonin-coated lactoferrin-chitosan nanoparticles	• Ionic gelation and carbodiimide coupling Etoposide	• The nanoparticles were 208.7 nm in size, with a zeta potential of 30.77 mV and an EE of 82.45% • It demonstrated a pH-responsive and prolonged release, releasing 98.68% over 24 h at a pH of 5.5 It showed improved cellular uptake and notable cytotoxicity in HCT116 cells	• The nanoparticles showed significant tumor shrinkage and selective colon accumulation in a rat model of colorectal cancer induced by DMH. • It increased plasma circulation and bioavailability, improving pharmacokinetics It inhibited opportunistic pathogens in colon cancer (*Proteus mirabilis)* and improved GM in treated rats	Raval et al. (2025)
Zein	Zein/*Mesona chinensis* polysaccharide nanoparticles (CR-NPs)	• Antisolvent precipitation and water evaporation-based crosslinking Resveratrol and curcumin	• CR-NPs demonstrated remarkable encapsulation rates of >88% for both curcumin and resveratrol with a tiny particle size (∼180 nm) It exhibited exceptional GI stability and low drug release in the stomach and small intestine, allowing for greater transport of curcumin and resveratrol to the colon	• CR-NPs treatment significantly reduced DSS-induced weight loss, blood in the stool, colon shortening, and tissue damage • It improved intestinal barrier and oxidative stress levels in UC mice and altered cytokine expression and immunological homeostasis It balanced intestinal flora, increased SCFAs synthesis, and regulated arachidonic, linoleic, and tryptophan metabolism	Yang et al. (2024a)
Oyster peptide	Oyster peptide/chitosan oligosaccharide/iron complex	• TGase-induced glycosylation Iron	• The iron complex had a loose and irregularly layered structure Iron addition enlarged the particle size, compressed the structure, decreased the intensity of UV and fluorescence, and changed the morphology	• The iron complexes enhanced iron absorption and augmented blood zinc concentration • It reduced IDA-induced organ abnormalities, oxidative stress, and colonic inflammation It restored IDA-induced GM dysbiosis	Yu et al. (2025)
Spanish mackerel protein	Spanish mackerel protein/pectin conjugates-stabilized Pickering emulsions	• Electrostatic complexation and emulsification Curcumin	• The SMP/PEC 1:1-stabilized emulsion demonstrated superior recovery, high viscosity, and outstanding pH and thermal stability It stayed stable in the stomach and released curcumin mostly in the intestine	• The emulsion revealed beneficial effects in DSS-induced colitis mice It suppressed the expression of inflammatory factors, decreased intestinal permeability, enhanced the expression of intestinal mucosal tight junction proteins, and restored GM composition	Sun et al. (2025)

More advanced designs further highlight gelatin’s potential in transporters. Li et al. found that incorporating hydrophilic gelatin into ethyl cellulose nanofibers improved quercetin release during colonic fermentation while maintaining protection during GI transit. Both free and encapsulated quercetin suppressed HCT-116 colon cancer cell proliferation via apoptosis induction, confirming the therapeutic potential of gelatin-based nanofibers, although *in vivo* validation remains necessary (Huang et al., 2023). Gelatin has also been used in multilayer structures with hyaluronic acid or sodium phytate to protect probiotics, although most evidence remains limited to *in vitro* studies (Dadmehr et al., 2024; Wang L. et al., 2024).

A recent study discovered a “Janus” structured nanoclay microgel employing microfluidic technology to target intestinal mucosal colonization with *Pediococcus pentosaceus* Li05, in which gelatin and alginate were used as the backbone polymers (Wu et al., 2025). In this study, polydopamine nanoparticles (PDA) were introduced into the black side of microgel to improve mucosal adherence and extend their colonic residence time. Nanoclays containing *P. pentosaceus* Li05 were assembled into the gray side of this gel to achieve strong adhesion and allow probiotic aggregation and proliferation within the microgel. *In vitro* findings showed that PDA allowed the PDA sides of microgels to align with the intestinal wall, facilitating their adherence to the mucosa. This longer residency period enhanced the effect of nanoclay and probiotics, increasing their therapeutic efficiency (Wu et al., 2025). The intestinal mucosa is a viscoelastic hydrogel made up of mucins and other proteins that are high in amino and carboxyl groups (Donahue et al., 2023). In contrast, PDA has rich catechol groups that attach to the carboxyl groups in intestinal proteins, as well as carboxyl groups that bind to the amino and positively charged groups in mucins, increasing their adherence to the intestinal mucosa. Furthermore, PDA can be attached to the cell surface via hydrogen or covalent bonds due to the presence of intrinsic amine, thiol, and hydroxyl groups on cell membranes (Pan et al., 2018). It was also observed that nanoclays stimulated bacteria aggregation and development due to their high cell compatibility and negative surface charge, causing *P. pentosaceus* Li05 to aggregate and stick to the clay (Wu et al., 2025). During their stay in the intestinal mucosa, microgel networks may slowly collapse owing to intestinal pH and enzymes, leading to the release of PDA and nanoclay, followed by the release of *P. pentosaceus* Li05 cells. Taken together, the Janus microgel design provides a dual-function platform that enhances mucosal adhesion and prolongs probiotic retention, thereby offering superior therapeutic efficiency compared to conventional single-component delivery systems.

Similarly, gelatin has been employed in responsive delivery systems (Bai et al., 2025). Reactive oxygen species (ROS)-sensitive dopamine-coated gelatin nanoparticles (Gel/DA-NPs) effectively delivered quercetin and alleviated colitis in DSS-induced mice by improving epithelial morphology, reducing apoptosis, promoting proliferation, regulating inflammatory cytokines, and inducing M2 macrophage polarization. These findings demonstrated the potential of quercetin-loaded Gel/DA-NPs for the treatment of ulcerative colitis (UC) and underscore the need for further preclinical validation to facilitate potential clinical translation (Bai et al., 2025). Similarly, a supramolecular liposome-microgel complex (SLMC) incorporated with *Bacillus* subtilis spores (BSSs) and dexamethasone (DEX) was reported as an orally deliverable probiotic UC therapy. *In vivo* studies demonstrated that SLMC therapy restored gut homeostasis by increasing the number of *Firmicutes* and *Lactobacillus* while decreasing the quantity of toxic *Proteobacteria*. The system further promoted tryptophan metabolite release and contributed to UC mitigation. Additionally, the SLMC system may release DEX to block the TLR4-NF-κB pathway, resulting in accelerating disease recovery (Fu et al., 2025). This study described a way to establish effective probiotic UC therapy with high recipient compliance.

## 4 Casein-based transporters

### 4.1 Chemical and functional properties of casein

Casein, a family of related phosphoproteins present in mammalian milk, has amphiphilic properties that enable it to form micelles and function as a natural emulsifier. It is relatively unstructured and flexible, with a high proline content and few secondary structures, such as alpha-helices or beta-sheets. Thus, it is classified as αS1-casein, αS2-casein, β-casein, and κ-casein, each contributing distinct roles such as calcium binding or micelle stabilization (Zhu et al., 2020; Raak and Corredig, 2022). Casein is poorly soluble in water but can disperse in dilute alkalis and certain salt solutions. The PI of casein is around pH 4.6, which means it forms a gel or clot in the stomach, resulting in a slow release of amino acids (Raak and Corredig, 2022). But it is digested more slowly than whey protein (Mulet-Cabero et al., 2020). Despite susceptibility to degradation under pH fluctuations, mineral imbalance, high temperature, or enzymatic action, casein is widely used in food, pharmaceutical, and healthcare applications. Recent studies have proposed innovative strategies to circumvent the constraints of casein, notably in drug delivery (Lin et al., 2024; Qian et al., 2025).

### 4.2 Delivery performance and gut health improvements of casein-based transporters

A recent study observed that complexing lactoferrin with β-casein (at a 2:1 ratio) enhanced protein stability under gastric conditions, resulting in improved intestinal transport and greater bioactivity (van der Schaaf et al., 2024). Such complexes may offer an effective strategy for stabilizing labile compounds, though further studies are needed to confirm targeted delivery and therapeutic potential. Li et al. used a pH-driven self-assembly approach to create two soft assembly systems to treat DSS-induced UC: curcumin/egg white peptide aggregates (Cur/EWP) and curcumin/egg white peptide/casein/quaternary chitosan complexes (Cur/EWP/CA/QC) (Li Y. et al., 2024). Both nanocomplexes demonstrated outstanding colloidal stability and biocompatibility, forming uniform spherical particles (156–300 nm). Curcumin retention after simulated intestinal digestion was ∼66.4% in Cur/EWP and ∼86.4% in Cur/EWP/CA/QC, indicating superior protection. The biodistribution investigation showed prolonged GI residence and stronger mucosal adherence for Cur/EWP/CA/QC, enabling sustained release and enhanced systemic absorption (Li Y. et al., 2024). *In vivo*, Cur/EWP/CA/QC demonstrated synergistic therapeutic efficacy by reducing colon shortening, disease activity index, and histological damage, while suppressing pro-inflammatory cytokines (TNF-α, IL-1β, IL-6) and increasing IL-10. It also enhanced intestinal permeability of curcumin and peptides, stimulated immune responses, upregulated tight junction proteins (ZO-1, MUC-2), enriched beneficial bacteria (*Akkermansiaceae, Lactobacillaceae*), and boosted SCFA production. Even in the unabsorbed stage, it promoted favorable microbiota growth, further contributing to anti-inflammatory effects (Li Y. et al., 2024). This study proposed a unique strategy for creating functional nanomaterials with a synergistic impact on UC relief via the soft assembly of hydrophilic active peptides and hydrophobic polyphenols.

Beyond polyphenols, casein phosphopeptides (CPPs) have also demonstrated promise as nutraceutical carriers. Zhu et al. created CPP–chitosan oligosaccharide systems via Amadori-type and TGase-catalyzed reactions, which enhanced calcium absorption in Caco-2 cells, modulated GM composition, and increased microbial metabolites such as butyrate (Zhu et al., 2020). Similarly, *Saccharomyces boulardii* (SB), a probiotic used for diarrhea and IBD, achieved improved GI stability and antioxidant activity when coated with tannic acid/Mg^2+^ complexes followed by CPP. In DSS-induced colitis models, SB-loaded CPP composites reduced epithelial damage and inflammation while enriching beneficial taxa (*Muribaculaceae, Lactobacillus, Bifidobacterium*) and suppressing pathogenic genera (*Alistipes, Escherichia-Shigella*). Enhanced tight junction protein expression (ZO-1, occludin) and reduced inflammatory cytokines further supported their therapeutic relevance (Qian et al., 2025). Casein-based systems have also improved the stability and delivery of polyphenols with poor bioavailability (Ma J. et al., 2025). For instance, Li et al. encapsulated EGCG into casein/sulfobutylether-β-cyclodextrin nanoparticles (TCNPs), which achieved high encapsulation efficiency (95%), strong antioxidant activity, and sustained release under simulated GI conditions. These results encourage further pharmacokinetic and pharmacological evaluation (Li H. et al., 2025).

Simvastatin (SV) is a poorly water-soluble and orally bioavailable statin drug, used to control cholesterol and prevent cardiovascular diseases. SV-loaded casein-stabilized solid self-emulsifying carrier (SV/Cas/SSE) was developed by ultrasonication and spray-drying techniques for improved GI delivery. Results demonstrated that SV/Cas/SSE displayed good stability, adequate redissolution capacity, and improved oral bioavailability of SV (Li H. et al., 2024). Therefore, the casein-stabilized solid self-emulsifying carrier could be a suitable transporter for poorly oral bioavailable drugs that have a promising effect on gut health, but sufficient research is required in this area. Resveratrol and its derivatives can alleviate UC by improving the intestinal barrier, regulating GM, and targeting inflammatory signaling pathways (Li M. et al., 2022). Similarly, sodium caseinate/decylglycerol monooleate emulsions stabilized resveratrol, markedly enhancing its bioavailability (3.1-fold increase) and selectively enriching SCFA-producing bacteria such as *Blautia* (Lin et al., 2024). Likewise, casein/gellan gum (CS/GG)-stabilized LBP-loaded oil-in-water emulsion displayed greater LBP viability during storage, pasteurization, and GI digestion compared to CS emulsion (Zhang et al., 2025). Therefore, casein-based advanced transporters are capable of preserving and delivering active ingredients to the gut, favorably altering the gut microenvironment.

## 5 Whey protein-based transporters

### 5.1 Chemical and functional properties of whey protein

Whey protein (WP) is a milk-derived dietary supplement produced as a by-product during cheese production. WP typically consists of β-lactoglobulin (40%–50%), α-lactalbumin (12%–15%), immunoglobulins (8%), and bovine serum albumin (5%) (Zhang S. et al., 2021; Chaiwong et al., 2025). The β-lactoglobulin (18 kDa, 162 amino acid residues) belongs to the lipocalin family and can bind with small hydrophobic molecules, e.g., fatty acids, polyphenols, vitamins, etc., in the internal cavity of its β-barrel. On the other hand, α-lactalbumin (14 kDa, 123 amino acid residues) contains a large alpha-helical domain and a small beta-sheet domain. It may function as a carrier of hydrophobic lipids, vitamins, and metabolites (Wang et al., 2019). WP possesses good biocompatibility and excellent emulsifying, gelling, and encapsulation properties (Kan et al., 2023). Moreover, it acts as an antioxidant, immune regulator, pathogenic bacterial inhibitor, and positive modulator of GM (Zhang S. et al., 2021; Kan et al., 2023). Unfortunately, WP exhibits poor solubility and less functional characteristics near its PI or at elevated temperatures. It is sensitive to thermal treatments, pH variations, salts, and digestive enzymes, restricting its extensive application (Kan et al., 2023; Wang F. et al., 2024; Chaiwong et al., 2025). However, polymeric conjugation between WP and biopolymers via electrostatic interactions (Luo et al., 2022; Mao et al., 2024), glycosylation (Wang F. et al., 2024), Maillard reaction (Kan et al., 2023), and wet-heating treatment (Chaiwong et al., 2025) could improve the physicochemical stability and functionality of WP. Three forms of WP are available in the market; (i) whey protein concentrate (WPC, containing about 70%–80% protein with some lactose and fat) (Zhang S. et al., 2021; Wang F. et al., 2024), (ii) whey protein isolate (WPI, containing about 90% protein with lower lactose and fat) (Kan et al., 2023; Mao et al., 2024), and (iii) whey protein hydrolysate (WPH, enzymatically hydrolyzed form of WP that contained >90% protein) (Zou et al., 2023).

### 5.2 Delivery performance and gut health improvements of whey protein-based transporters

Accumulating evidence indicates that WP and its derivatives are promising carriers for colon-specific delivery of bioactives, and they may also act synergistically with encapsulated compounds to enhance gut health. For example, the complexation of cobalamin (vitamin B12), a thermo- and photosensitive molecule, with β-lactoglobulin or α-lactalbumin improved its stability by 10%–30%. Under simulated gastric digestion, stability increased by 19.7% for adenosylcobalamin and 2.2% for cyanocobalamin, while colonic digestion studies demonstrated enhanced bioavailability and favorable modulation of GM, including increased *Bacteroidetes* and *Firmicutes* and reduced *Proteobacteria* (Wang et al., 2019). Similarly, WP/fructooligosaccharide microcapsules were successfully developed for anthocyanin delivery from black soybean skin. These microcapsules exhibited greater antioxidant activity, gastrointestinal stability, and fermentation potential. They also promoted the growth of *Faecalibacterium* and *Akkermansia* while stimulating SCFA production (notably acetate and propionate), collectively contributing to improved intestinal health (Li X. et al., 2024). Even though this study indicated the potential of anthocyanin-microcapsules in modulating GM, more research is needed to confirm their efficacy in improving gut health.

Likewise, WPI/galacto-oligosaccharide (WPI/GOS) conjugates not only repaired intestinal barrier function and immune organ integrity in immunosuppressed mice but also modulated GM composition, enriched *Lactobacillus*, and activated NFκB-mediated immune responses. Histological analyses confirmed restoration of villus/crypt architecture, further supporting their therapeutic potential (Kan et al., 2023). Additionally, WPI/Tremella fuciformis polysaccharide-stabilized emulsions were developed for magnolol, a compound with therapeutic potential against ulcerative colitis (UC). This formulation enhanced magnolol stability, colonic delivery, and bioadhesion, while reducing DSS-induced colitis symptoms, preserving epithelial structure, and modulating GM composition in favor of *Lactobacillaceae* and *Firmicutes* (Zhang et al., 2024).

Additionally, Luo et al. introduced alginate (ALG)/WPI and ALG/pectin/WPI bead systems for the protection and delivery of probiotic *Lactobacillus plantarum* (LP). Both systems exerted excellent EE (>99%), protected LP during simulated gastric digestion, and released LP in the simulated intestinal phase (Luo et al., 2022). Moreover, encapsulation of *Lactiplantibacillus plantarum* (LBP) using WPI fibrils and multilayer polysaccharide microcapsules improved survival (90.16% after freeze-drying), gastrointestinal stability, and adhesion to colonic cells by 8.5-fold compared with uncoated fibrils (Li S. et al., 2025). Electrospray microcapsules prepared from WPC with polysaccharides (e.g., HPMC, dextran, trehalose, β-cyclodextrin) provided further protection under environmental stresses. Co-delivery with EGCG as a prebiotic enhanced both probiotic viability and antioxidant activity, with WPC/β-cyclodextrin microcapsules offering the greatest stability (Ma J. et al., 2025). Overall, these findings demonstrate that WP-based delivery systems can protect labile bioactives, improve stability and bioavailability, and modulate GM composition toward health-promoting profiles.

## 6 Soy protein-based transporters

### 6.1 Chemical and functional properties of soy protein

Soy protein is a popular plant-based protein derived from soybeans, which is regarded as the only “complete protein” among plant proteins, comprising all of the essential amino acids for human body development. Soy protein concentrate (SPC) and soy protein isolate (SPI) are two types of soy protein, which are composed of two key globulins, e.g., glycinin (11S) and β-conglycinin (7S), accounting for >70% of their total protein content (Yang Z. et al., 2024). They are rich in hydrophilic and hydrophobic amino acid residues, allowing various molecular interactions required for encapsulation. Glycinin (11S), which makes up ∼30% of soy protein, has a compact structure supported by disulfide bonds, electrostatic interactions, and hydrophobic forces. It produces nanogels upon heating, self-assembles into nanoparticles under specific circumstances, and forms stable complexes with vitamin B12, increasing its bioavailability (Sirison et al., 2021; Gong et al., 2024). β-conglycinin (7S) is a trimeric soy protein (∼180 kDa) composed of α, α′, and β subunits with hydrophobic regions that allow effective encapsulation of hydrophobic bioactives. Its quaternary structure is highly sensitive, dissociating into subunits under heat or urea treatment before reassembling into nanoparticles during dialysis. 7S forms stable nanoparticles containing beneficial substances like vitamin D_3_ and curcumin, enhancing their water solubility, thermal, light, and oxidative stability, and bioavailability (Li J. et al., 2022; Gong et al., 2024). Soy protein has strong emulsifying, gelling, and film-forming capabilities, making it ideal for creating micro- and nanocarriers. SPI’s amphiphilic nature enables it to encapsulate and distribute various bioactive compounds. Its capacity to undergo heat-induced denaturation, cross-linking, and pH-responsive structural changes promotes its use in controlled-release transporters. Furthermore, electrostatic and hydrogen bonding interactions between soy protein and active chemicals or polysaccharide partners help construct robust transporters (He et al., 2022; Gong et al., 2024). These characteristics could make soy protein an excellent plant-based choice for developing advanced transporters for natural GM modulators.

### 6.2 Delivery performance and gut health improvements of soy protein-based transporters

SPI effectively enhances gastrointestinal delivery of polyphenols while favorably modulating the GM. Maillard-type glycosylation of SPI improved redispersibility, stress stability, DPPH scavenging, and bioaccessibility of tea polyphenols; resultant nanoparticles increased *Bacteroides* and lowered the *Firmicutes/Bacteroides* ratio, indicating potential intestinal benefits (Yang et al., 2025). Soybean lipoprotein nanoparticles (SLP) loaded with curcumin (EE 77.13%) improved curcumin bioaccessibility (76.14% in the intestinal phase) and *in vivo* increased *Bifidobacterium* while reducing the *Firmicutes/Bacteroidetes* ratio, supporting Cur-SLP for intestinal health and as a candidate for IBD management (Zeng et al., 2023).

Recently, Zhao et al. introduced a highly adhesive bilayer microcapsule for the colonic delivery of LBP KLDS1.0386 (LP) and alleviation of DSS-induced IBD, where individual LP cells were modified by a tannic acid-Fe^3+^ (TA-Fe) nanocoating and then with soybean isolate protein/xanthan gum composite layer (SX) via hydrogen bonding (Zhao et al., 2025). The SX layer protected LP from gastric insult, while the TA-Fe layer promoted colonic colonization. In DSS-colitis, LP@TA-Fe/SX preserved epithelial architecture and crypt morphology, downregulated pro-inflammatory cytokines (IL-1β, IL-6, IL-8, TNF-α, IFN-γ), increased IL-10, and restored barrier markers (ZO-1, occludin-1, MUC2) (Yao et al., 2022; Li M. et al., 2024; Huang et al., 2025). At the genus level, *Escherichia–Shigella* was suppressed, while the SCFA-producing, anti-inflammatory *Lachnospiraceae_NK4A136_group* increased.

Soy proteins have also been engineered into hybrid delivery systems. Han et al. combined soybean 7S protein (7S) with dietary calcium phosphate (CaP) to develop a 7S/CaP probiotic tablet co-delivering *Lactobacillus rhamnosus* (Han et al., 2019; Qiu et al., 2022). This formulation protected probiotics from gastric stress, enabled controlled intestinal release, improved colonic delivery, restored mucus secretion, reinforced tight junctions (occludin, ZO-1), and enriched beneficial bacteria (genus *Lacticaseibacillus*, species *L. rhamnosus*), thereby alleviating colitis in DSS mice (Han et al., 2025). Likewise, encapsulation of *Lactobacillus delbrueckii* in pectin–soy protein hydrogels ameliorated antibiotic-induced diarrhea, weight loss, and depression-like behaviors, effects associated with increased colonic colonization by *Lactobacillus* and *Bifidobacterium* (Sun et al., 2015; Jiang et al., 2022). Soy hull polysaccharide/SPI (SHP/SPI)-based high internal phase emulsions (HIPEs) have also emerged as promising probiotic carriers. At 1.8% SHP, HIPEs displayed reduced droplet size, improved structural stability, and high encapsulation efficiency (99.3%), achieving delivery efficiency of 71.1%. Both *in vitro* and *in vivo* studies confirmed improved probiotic survival and colonization, with notable post-pasteurization viability, underscoring their potential for colon-targeted probiotic delivery in functional foods (Sun et al., 2024).

Although soy proteins have demonstrated great promise in improving the stability and bioactivity of natural GM modulators, their functional performance is affected by poor solubility, suboptimal emulsifying and rheological properties, and conformational heterogeneity. SPI-based nanoparticles often suffer from poor size uniformity, low preparation efficiency, and limited scalability beyond the laboratory setting (Gong et al., 2024). Allergenicity further restricts application in sensitive populations. Moreover, heat-induced gels formed by soy proteins typically display low mechanical strength, limiting their broader use in food and nutraceutical applications (Liu et al., 2024).

## 7 Zein-based transporters

### 7.1 Chemical and functional properties of zein

Zein is a plant protein derived from the endosperm of maize kernels and is a member of the prolamin class. It is amphiphilic because of its distinct molecular structure and amino acid composition, which includes both nonpolar amino acids (hydrophobic), such as leucine (20%), proline (10%), and alanine (12%), as well as polar amino acids (hydrophilic) like glutamine (∼20%). Consequently, it is insoluble in water but soluble in aqueous ethanol (>70%) (Tadele et al., 2025). Zein prolamins are classified into four categories based on solubility and structure: α-, β-, γ-, and δ-zein prolamin. Of them, the α-zein prolamin, with a molecular weight of 19–22 kDa, comprises 75%–85% of the total composition (Tadele et al., 2025). Apart from amino acid composition, zein characteristics might vary depending on their molecular structures. Many models of zein’s structure have been offered, including Agros’ cylinder model, Garrett’s wheel model, Matasushima’s elongated model, Fofato’s hairpin model, and Bugs’ supercoiled structure model. Although these models are inconclusive, there is broad agreement regarding the linear stacking of rod-shaped helical repeat units (Sun et al., 2023). The solubility of zein is affected by protein concentration, pH, temperature, and ion strength (Wei et al., 2019). Hydrolysis and glycosylation have been described as useful techniques for improving zein’s water solubility. It has an amorphous structure with a glass transition temperature of 165 °C and strong thermal stability of up to 280 °C (Sun et al., 2023). Zein possesses outstanding film-forming, emulsifying, stabilizing, and self-assembly characteristics. Due to the PI around pH 6.8, it can show positive charges at pH < 6.0 and negative charges at pH > 7.0, and promote electrostatic interactions in the mixture. Therefore, it is applied to form complexes with a variety of polysaccharides, including chitosan, sodium alginate, xanthan gum, and pectin, for the protection and oral delivery of sensitive molecules (Sun et al., 2023; Tadele et al., 2025). Zein-based transporters, as an alternative to animal protein, have lately received interest in the food and nutraceutical industries due to their non-toxicity, biocompatibility, biodegradability, and contribution to targeted-release properties (Tadele et al., 2025). In summary, it is undeniable that zein’s chemical characteristics lay the groundwork for developing transporters, and its functional characteristics may dictate how well it might preserve active ingredients, regulate release, and support gut health.

### 7.2 Delivery performance and gut health improvements of zein-based transporters

Zein readily forms nanoparticles through methods such as desolvation and electrospinning, making it a promising excipient for colon-targeted delivery. For example, quercetin-loaded zein nanospheres (NS) and nanocapsules (NC) exhibited similar physicochemical properties, yet NS showed superior intestinal interaction and significantly enhanced quercetin bioavailability in rats (57% vs. 26% for NC, >10-fold higher than controls) (Campión et al., 2023). Likewise, zein-succinic acid-modified cyclodextrin nanofibers improved curcumin stability and enabled targeted colonic release with minimal release (5.9%–15.7%) in the upper GI tract (Hu et al., 2024). Similarly, coating zein nanoparticles with *Mesona chinensis* polysaccharide further enhanced curcumin bioavailability and bioactivity, including ROS-mediated tumor cell apoptosis (Yang et al., 2022). Other *in vitro* studies demonstrated similar efficacy for zein/GA nanocarriers delivering seabuckthorn flavonols (Sheng et al., 2025) and zein/flaxseed gum oligosaccharides for apigenin (Liu et al., 2025b), warranting further *in vivo* validation.

Zein-polysaccharide transporters have also been developed for probiotic delivery (Lei et al., 2024). Zein/soluble soybean polysaccharide nanoparticles (ZPS) improved the viability of *Bacillus subtilis* by > 3-fold under simulated digestion, pasteurization, and storage (Cheng et al., 2024). Further, ZPS-coated probiotics reduced potentially harmful bacterial populations (*Escherichia-shigella*) while simultaneously improving microbial diversity, GM composition, and the abundance of beneficial bacteria (*Muribaculaceae, Romboutsia, Bifidobacterium,* and *Lactobacillus*) (Cheng et al., 2024). *Muribaculaceae*, a bacterial family under the order *Bacteroidetes*, have a positive role in sustaining host health by generating SCFAs and regulating intestinal barrier function and immunological response (Zhu et al., 2024). Similarly, as components of the GM, *Bifidobacterium* and *Lactobacillus* may have a favorable role in antagonizing harmful bacteria and alleviating IBD (Alonso-Roman et al., 2022; Gao et al., 2024). Additionally, *Romboutsia lituseburensis* supplementation has been linked to improved endothelial function through GM regulation (Yin et al., 2023).

Zein-based systems have demonstrated disease-modifying potential in mouse models. Zein/Mesona chinensis polysaccharides amorphous nanoparticles (CR-NPs) were synthesized using antisolvent precipitation and water evaporation-based crosslinking to deliver resveratrol and curcumin together for the targeted treatment of DSS-induced UC (Yang J. et al., 2024). The symptoms of DSS-induced weight loss, blood in the stool, colon shortening, mucosal injury, disturbed crypt structure, and intestinal inflammation were all markedly reduced by CR-NPs (Yang J. et al., 2024). At the genus level, it increased the abundance of *Ruminococcus, Oscillospira,* and *Coprococcus*, which are beneficial bacteria for SCFAs production, while reducing the abundance of harmful bacteria, including *Shigella, Clostridium,* and *Sutterella*. Subsequently, it increased the synthesis of SCFAs such as acetic acid, propionic acid, butyric acid, and pentanoic acid while also modulating arachidonic acid, linoleic acid, and tryptophan metabolism (Yang J. et al., 2024). Similarly, egg-white peptide–zein/chondroitin sulfate nanoparticles enhanced quercetin’s colon-targeting, promoted tight junction repair, and enriched beneficial bacteria (*Lachnospiraceae, Muribaculu*m, *Akkermansia*) while suppressing pathogenic taxa (Ma S. et al., 2025). Collectively, these studies highlight zein as a versatile platform for delivering polyphenols, probiotics, and other bioactives with gut-targeted functionality. However, very few *in vivo* studies have investigated the potential of zein-based transporters for improving gut health. More research is needed to synthesize zein-based robust systems and verify their therapeutic benefits through well-designed *in vivo* experiments and human trials.

## 8 Other protein-based transporters

Numerous studies have sought to investigate alternative proteins for the fabrication of advanced oral transporters. Only a handful of these have been turned into *in vivo* applications. Oyster peptide and Spanish mackerel protein are two proteins that have recently been used to create advanced transporters for *in vivo* use (Yu et al., 2023; Sun et al., 2025; Yu et al., 2025). In DSS-induced colitis mice, the Spanish mackerel protein/pectin conjugates-stabilized Pickering emulsions encapsulating curcumin showed positive effects by controlling the expression of inflammatory factors, decreasing intestinal permeability, increasing the expression of intestinal mucosal tight junction proteins, and improving microbiota dysbiosis (Sun et al., 2025). Likewise, a mouse model of iron deficiency anemia (IDA) was used to examine the impact of the oyster peptide/chitosan oligosaccharide/iron complex. Oral administration of iron complexes improved iron absorption, increased blood zinc content, decreased IDA-induced organ abnormalities, oxidative stress, and colonic inflammation, and restored IDA-induced GM dysbiosis (Yu et al., 2025). *In vivo* investigations on protein-based transporters revealed encouraging results in terms of lowering oxidative stress, gut inflammation, and GM disorders. However, some of them may not be viable for commercial production due to the multiple-step synthesis procedure, long synthesis time, and rigorous reaction conditions necessary for fabrication. More research is advised before clinical use to streamline the manufacturing process without compromising the targeted and functional qualities of the transporters.

## 9 Current challenges and possible solutions of protein-based transporters

Protein-based transporters encounter significant challenges, which limit their therapeutic efficacy and clinical application. The major obstacles are outlined as follows:

• Process control: Proteins are prone to denaturation or breakdown due to high mechanical stress, pH changes, and temperature fluctuations. Additionally, they could have unfavorable structural and functional alterations during storage and processing. Therefore, gentle processing conditions should be maintained throughout the manufacturing process of protein-based transporters.
• GI instability: Protein-based nanoparticles are very susceptible to enzymatic degradation, and they may aggregate at pH values around their PI or in high ionic strength, necessitating additional modification for stability. For example, zein and PPI nanoparticles were stabilized by WPI/carboxymethyl cellulose and fucoidan-quaternary ammonium chitosan complex, respectively, which not only enhanced the carrier stability but also improved the controlled-release and targeted delivery of apigenin and pterostilbene. Furthermore, the rapid clearance of transporters from the circulatory system may induce therapeutic failure owing to inadequate mucosal permeability, glomerular filtration, phagocytosis, and non-specific targeting, which must be considered during research design.
• Immunogenicity: Some carrier polymers, e.g., poly-lactic acid and poly-lactic-co-glycolic acid, may activate unexpected immunological responses and generate inflammatory byproducts, which can impair therapeutic efficacy and health safety (Wu et al., 2022). The spontaneous glycation of whey protein during industrial/thermal processing and storage may boost immunogenic responses (Złotkowska et al., 2023). Likewise, digestion-resistant epitopes, derived from egg white, milk, and wheat gluten proteins, may pose an allergen risk. Therefore, a protein-based formulation with no/low toxicity risks should be implemented in the transporters.
• Reproducibility: Reproducibility challenges arise during large-scale production of protein-based transporters because variations in protein properties (e.g., molecular weight, purity, stability, etc.) can negatively affect the functional properties and delivery performances of transporters. Therefore, it necessitates precise control over manufacturing, formulation, and storage, along with comprehensive characterization using cutting-edge analytical techniques. Process optimization and a standardized stabilization strategy are required to improve repeatability and scale-up operations.

## 10 Commercial potential of protein-based transporters

Protein-based delivery vehicles have attracted substantial academic attention over the past decade and demonstrated tremendous commercial promise. The potential commercial prospects are summarized below:

• Functional food products: Protein-based transporters can enhance the stability, bioavailability, and controlled/pH-responsive release of nutraceuticals and/or probiotics. Therefore, they could address key challenges in the development of functional and fortified food items. For example, protein nano-complexes or hydrogels encapsulating functional ingredients can be introduced into yogurts, beverages, and bakery foods, which could protect the active ingredients during processing and storage, without affecting the product’s taste or texture.
• Precise delivery of nutrients: Protein carriers provide a platform for designing personalized dietary formulations, such as low-glycemic snacks for diabetic patients, probiotic-enriched foods for maintaining gut health, and performance-oriented protein bars for athletes.
• Target-specific delivery: Owing to their ability to interact with specific tissues and cells, protein-based transporters hold promise for treating gut disorders and metabolic diseases, and can be combined with prebiotic-probiotic strategies to achieve synergistic effects.
• Commercial application: To date, the majority of protein-based transporters for gut disorders are still in the preclinical phase. Only a few albumin-based therapeutics like the chemotherapy drug “Abraxane^®^,” which contains albumin nanoparticles/paclitaxel, and some albumin-binding medications, such as “Ibuprofen” (anti-inflammatory), “Warfarin” (anticoagulant), and “Exenatide” (anti-hyperglycemic agent), have been approved for the treatment of metabolic disorders (Murphy et al., 2025). The commercial availability of such therapeutics illustrates the viability of protein-based transporters. Moreover, ongoing investment in protein-based therapeutics is expected to boost market expansion and broaden clinical applications.

## 11 Conclusions and future directions

This review demonstrates that protein-based micro- and nano-transporters constitute a versatile and promising platform for the delivery of natural GM modulators. Their intrinsic biodegradability, biocompatibility, tunable particle size, facile surface functionalization, and capacity to protect labile cargos from acidic and enzymatic degradation enable enhanced stability, targeted release, and improved bioavailability of polyphenols, peptides, and probiotics. Preclinical studies consistently indicate that such carriers can mitigate dysbiosis, promote beneficial microbial taxa, increase short-chain fatty acid production, strengthen epithelial barrier integrity, and modulate mucosal immune responses.

Notwithstanding these advances, several critical barriers limit translational potential. Key technical challenges include physiological instability in complex gastrointestinal environments, insufficient strain- or tissue-specific targeting, potential immunogenicity, batch-to-batch variability, and limited long-term safety data. Moreover, heterogeneity in experimental characterization and outcome metrics hampers cross-study comparison and systematic assessment of efficacy. The adoption of emerging strategies, including rational surface engineering (mucoadhesive ligands, receptor-targeting motifs), protease- and acid-resistant peptide motifs, hybrid protein–polysaccharide assemblies, and stimuli-responsive designs, will help address these limitations. Furthermore, integration of advanced manufacturing (GMP-compatible processes, scalable encapsulation techniques), quantitative multi-omics and mechanistic studies (metagenomics, metabolomics, host transcriptomics), and standardized safety assessments will be essential for clinical translation. Computational tools and AI-guided design can accelerate optimization of carrier architecture and predict host–microbiome responses, while micro- and nanorobotics offer a long-term frontier for active, precision delivery.

In summary, protein-based transporters have strong potential to translate natural GM modulators into clinically relevant nutraceuticals and therapeutics. Future research should focus on carrier optimization, targeting specificity, and comprehensive clinical validation to fully realize the potential of these systems in gut health management.
